# Prediction and Risk Stratification of Cardiovascular Disease in Diabetic Kidney Disease Patients

**DOI:** 10.3389/fcvm.2022.923549

**Published:** 2022-06-24

**Authors:** Jingjing Ren, Dongwei Liu, Guangpu Li, Jiayu Duan, Jiancheng Dong, Zhangsuo Liu

**Affiliations:** ^1^Department of Integrated Traditional and Western Nephrology, The First Affiliated Hospital of Zhengzhou University, Zhengzhou, China; ^2^Research Institute of Nephrology, Zhengzhou University, Zhengzhou, China; ^3^Henan Province Research Center for Kidney Disease, Zhengzhou, China; ^4^Key Laboratory of Precision Diagnosis and Treatment for Chronic Kidney Disease in Henan Province, Zhengzhou, China; ^5^Clinical Research Center of Big-data, The First Affiliated Hospital of Zhengzhou University, Zhengzhou, China

**Keywords:** machine learning, diabetic kidney disease, cardiovascular disease, prediction model, risk stratification

## Abstract

**Background:**

Diabetic kidney disease (DKD) patients are facing an extremely high risk of cardiovascular disease (CVD), which is a major cause of death for DKD patients. We aimed to build a deep learning model to predict CVD risk among DKD patients and perform risk stratifying, which could help them perform early intervention and improve personal health management.

**Methods:**

A retrospective cohort study was conducted to assess the risk of the occurrence of composite cardiovascular disease, which includes coronary heart disease, cerebrovascular diseases, congestive heart failure, and peripheral artery disease, in DKD patients. A least absolute shrinkage and selection operator (LASSO) regression was used to perform the variable selection. A deep learning-based survival model called DeepSurv, based on a feed-forward neural network was developed to predict CVD risk among DKD patients. We compared the model performance with the conventional Cox proportional hazards (CPH) model and the Random survival forest (RSF) model using the concordance index (C-index), the area under the curve (AUC), and integrated Brier scores (IBS).

**Results:**

We recruited 890 patients diagnosed with DKD in this retrospective study. During a median follow-up of 10.4 months, there are 289 patients who sustained a subsequent CVD. Seven variables, including age, high density lipoprotein (HDL), hemoglobin (Hb), systolic blood pressure (SBP), smoking status, 24 h urinary protein excretion, and total cholesterol (TC), chosen by LASSO regression were used to develop the predictive model. The DeepSurv model showed the best performance, achieved a C-index of 0.767(95% confidence intervals [CI]: 0.717–0.817), AUC of 0.780(95%CI: 0.721–0.839), and IBS of 0.067 in the validation set. Then we used the cut-off value determined by ROC (receiver operating characteristic) curve to divide the patients into different risk groups. Moreover, the DeepSurv model was also applied to develop an online calculation tool for patients to conduct risk monitoring.

**Conclusion:**

A deep-learning-based predictive model using seven clinical variables can effectively predict CVD risk among DKD patients and perform risk stratification. An online calculator allows its easy implementation.

## Introduction

Diabetic kidney disease (DKD) has been one of the most serious diabetic microvascular complications, implicating up to 50% of patients with diabetes and becoming the major cause of the end-stage renal disease (ESRD) worldwide (1–3). DKD patients are at high risk of developing cardiovascular disease (CVD), bringing a heavy burden on the public health system (4, 5). Reduction in renal function is considered as an independent risk factor and predictor of CVD (6, 7). Therefore, DKD patients are more susceptible to CVD than the general population, resulting in much worse functional outcomes, morbidity, and mortality (8, 9). Meanwhile, they also have under-treated problems because of the lack of awareness of the CVD risks. However, the mechanisms by which CVD occurs in patients with DKD have also not been fully clarified. The individual performance and prognosis are often heterogeneous. Accordingly, improving awareness of cardiovascular risk factors and conducting early intervention in high-risk patients may improve prognosis and slow the progress.

There are many risk factors associated with the high prevalence of CVD in DKD patients. Studies of risk factors and predictive tools for CVD are common among the general population, such as the Framingham, QRISK, and China-PAR models (10–12). But these models often excluded the populations with a decline in kidney function, so they cannot be fully applied to the DKD patients with high risk of cardiovascular events (13, 14). Researches on cardiovascular risk factors in this population are still limited. Network Initiative Cardiovascular and Renal Clinical Trialists (INI-CRCT) also advise that we should attach more importance to these patients to enhance their cardiovascular outcomes (15). Therefore, identifying these cardiovascular risk factors and patients at high risk is still a challenging problem.

In recent years, artificial intelligence, particularly deep learning is developing rapidly and has been applied to a variety of medical fields, such as disease prediction (16), machine vision (17) and diagnostic study (18), etc. Deep learning is superior in handling different types of data for its strong computing power (19). So far, there have been many deep learning methods were developed for survival analysis (20, 21). Katzman et al. also developed a novel deep feed-forward neural network based on Cox assumption called DeepSurv, which combined survival analysis with deep learning and had the advantage to perform a prediction of time-to-event data. It has been successfully applied in the survival analysis of multiple diseases and showed promising performance in predicting patients' outcomes, such as oncological diseases, Covid-19, and atherosclerotic cardiovascular disease (22–26). Several online calculation tools were constructed based on the DeepSurv method (27–29).

In this study, we aimed to develop a predictive model based on the deep learning method to predict the progression of CVD in DKD patients. It also can help us to investigate the associated risk factors, to provide treatment recommendations for better cardiovascular outcomes and support personalized medicine. Patients were stratified into different risk subgroups using the output risk values from the model. We further evaluated the performance of the DeepSurv model with the classical Cox proportional hazards (CPH) model and the Random survival forest (RSF) model using the concordance index (C-index), to prove that machine learning can effectively improve the predictive performance. Additionally, an easy-used online tool for calculating the incidence rate of CVD in DKD patients based on the predictive model was developed.

## Materials and Methods

### Study Population

We retrospectively reviewed the patients who were diagnosed with DKD from the electronic medical records of the First Affiliated Hospital of Zhengzhou University from January 2013 to January 2020. DKD is defined as diabetic patients who performed persistent urinary albumin excretion or a reduction in estimated glomerular filtration rate (eGFR) for more than 3 months (2). Patients were required to have at least two hospitalizations, which allowed us to record patients' baseline data before a CV event (if any). The exclusion criteria were: (1) patients with incomplete clinical information (*n* = 17); (2) age <18 years (*n* = 1); (3) patients had history of cardiovascular diseases or coronary revascularization (*n* = 1,065); (4) patients with surgery, infection or injured at baseline (*n* = 18); and (5) patients with autoimmune disease or malignant tumor (*n* = 45). The selection process of patients is shown in Supplementary Figure 1.

### Cardiovascular Outcomes

The CVD outcomes in this study were the first occurrence of a subsequent CVD, including coronary heart disease (coronary heart disease, myocardial infarction, angina, and coronary revascularization); cerebrovascular disease (hemorrhagic stroke and ischaemic stroke); congestive heart failure and peripheral arterial disease (amputations, aortic aneurysm, revascularization of the aorta or other peripheral arteries) and the combination of cardiovascular events. Outcomes were defined by the International Classification of Diseases, Tenth Revision (ICD-10) codes. The ICD-10 codes are summarized in Supplemental Table 1.

### Statistical Analysis

we extracted the baseline patients' characteristics from the electronic medical records, including demographic details, comorbidities, physical examination measures, and laboratory values. If the missing-value ratio of a variable is more than 30%, the variable will be excluded. To impute the missing data, we applied multivariate imputation by chained equations (MICE) (30). After data imputation, we used the *Z*-score normalization method to normalize all variables to reduce the bias. The least absolute shrinkage and selection operator (LASSO) regression was performed to determine the significant clinical variables using 10-fold cross-validation (31). Furthermore, we used both univariate and multivariate cox regression analysis to assess the independent prognostic significance of the selected variables.

### Modeling Process

The database was divided into two mutually exclusive datasets with balanced data distribution, 70% as a training set and 30% as an internal validation set. Continuous variables are presented as mean ± standard deviation (SD) and compared between groups using the *t*-test, or as median (interquartile range [IQR]) and compared by Mann–Whitney *U-*tests, depending on whether the data is normally distributed or not. Categorical variables are shown as frequency (percentage) and compared by the chi-squared test. The cumulative incidence rate of the two sets was plotted by the Kaplan-Meier method and then compared using the log-rank test. Deep Learning-based Survival Model (DeepSurv) was used to perform the task of predicting patient-individual cardiovascular risk using the preselected variables by LASSO regression. We applied Bayesian hyperparameter optimization including learning rate decay, dropout, and other hyperparameters, to prevent overfitting (32). The list of hyperparameters of DeepSurv was provided in the Supplementary Table 2. More details about the DeepSurv method are available online (https://github.com/jaredleekatzman/DeepSurv). After splitting the datasets into the training and validation sets, we trained the model performing a 5-fold cross-validation on the training set to assess for overfitting and determine the model validity. Then we estimated the model performance in the validation set.

### Model Validation

**Figure 1:**
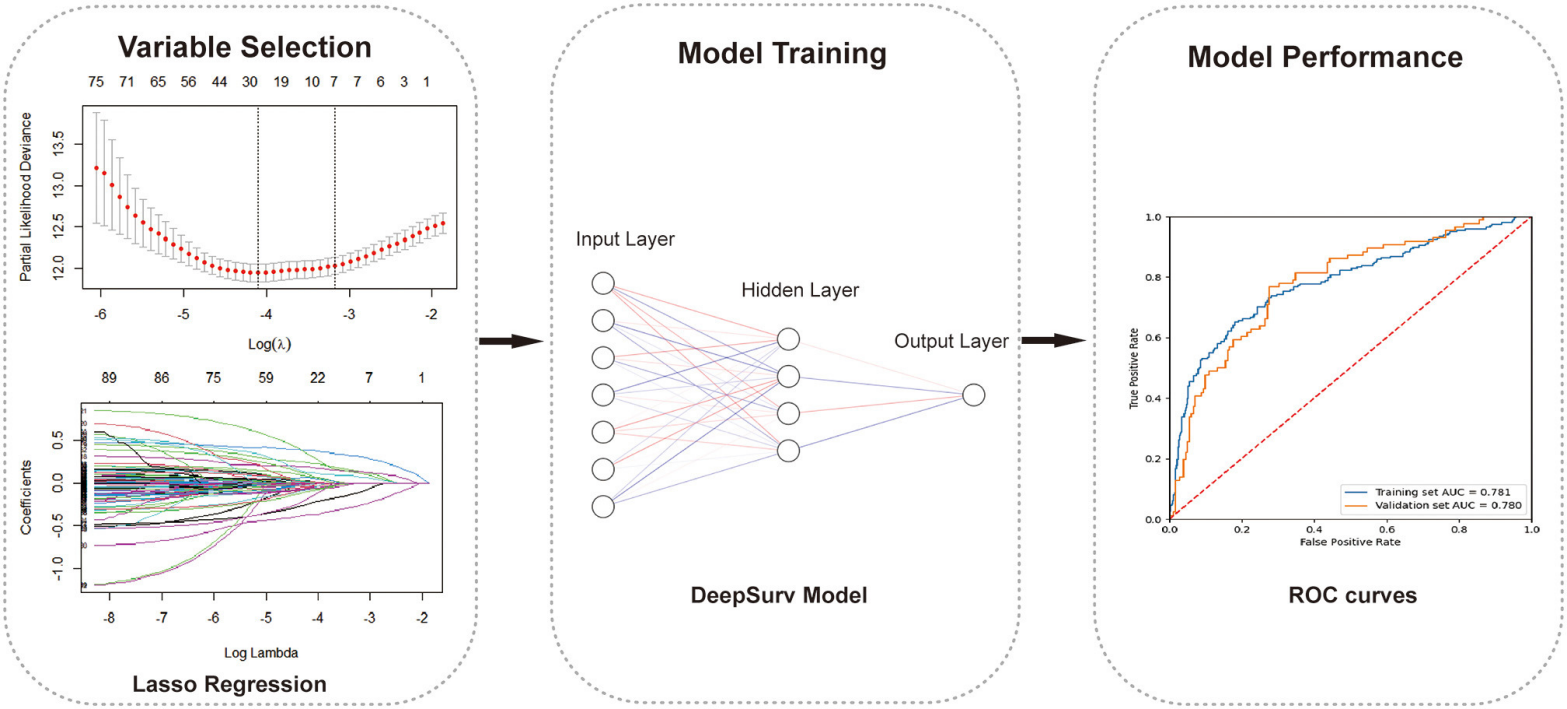
The working process of this study. The study procedure consisted of variables selection, model building, and model evaluation.

After obtaining the output predicted individual risks with the DeepSurv model, the DKD patients were then divided into high-risk and low-risk groups based on the cut-off value determined by ROC (receiver operating characteristic) curve. The cumulative incidence curves were plotted using the Kaplan-Meier method and compared using the log-rank test to visualize the difference in the predicted cumulative incidence of patients in two risk groups. The importance values of the selected variables were calculated by their component weights in the DeepSurv model, indicating the univariate contribution to the model. Finally, we compared the performance of the DeepSurv model with the CPH model and RSF model as conventional prediction methods to verify its performance. The discrimination performance of the proposed methods was assessed using the C-index, integrated Brier score (IBS) (33), and the area under the receiver-operator characteristic curve (AUC) in both the training and validation cohorts. C-index is a commonly used indicator of survival prediction, which can reflect the ability to predict time-to-event data. IBS compares the predicted survival rate with the actual status of patients. Higher C-index, higher AUC, and smaller IBS indicate a stronger fit of the model. Then we plotted the calibration curves of the predicted individual risk probabilities of CVD events at 1, 3, and 5 year. Furthermore, for these four different cardiovascular diseases included in our study, a subgroup analysis was conducted to determine which of their outcomes has the best-predicted performance using the DeepSurv models. A simple depiction of our study design is shown in Figure 1. The study was reported according to the Transparent Reporting of a multivariable prediction model for Individual Prognosis or Diagnosis (TRIPOD) guidelines (Supplementary Table 3). Statistical analysis was completed with R v.4.1.1 and SPSS v.26 (IBM Corporation) software. Python v.3.7 and TensorFlow v.1.14 were used to implement DeepSurv models. A *p*-value < 0.05 was considered statistically significant.

## Results

### Patient Characteristics

**Figure 2:**
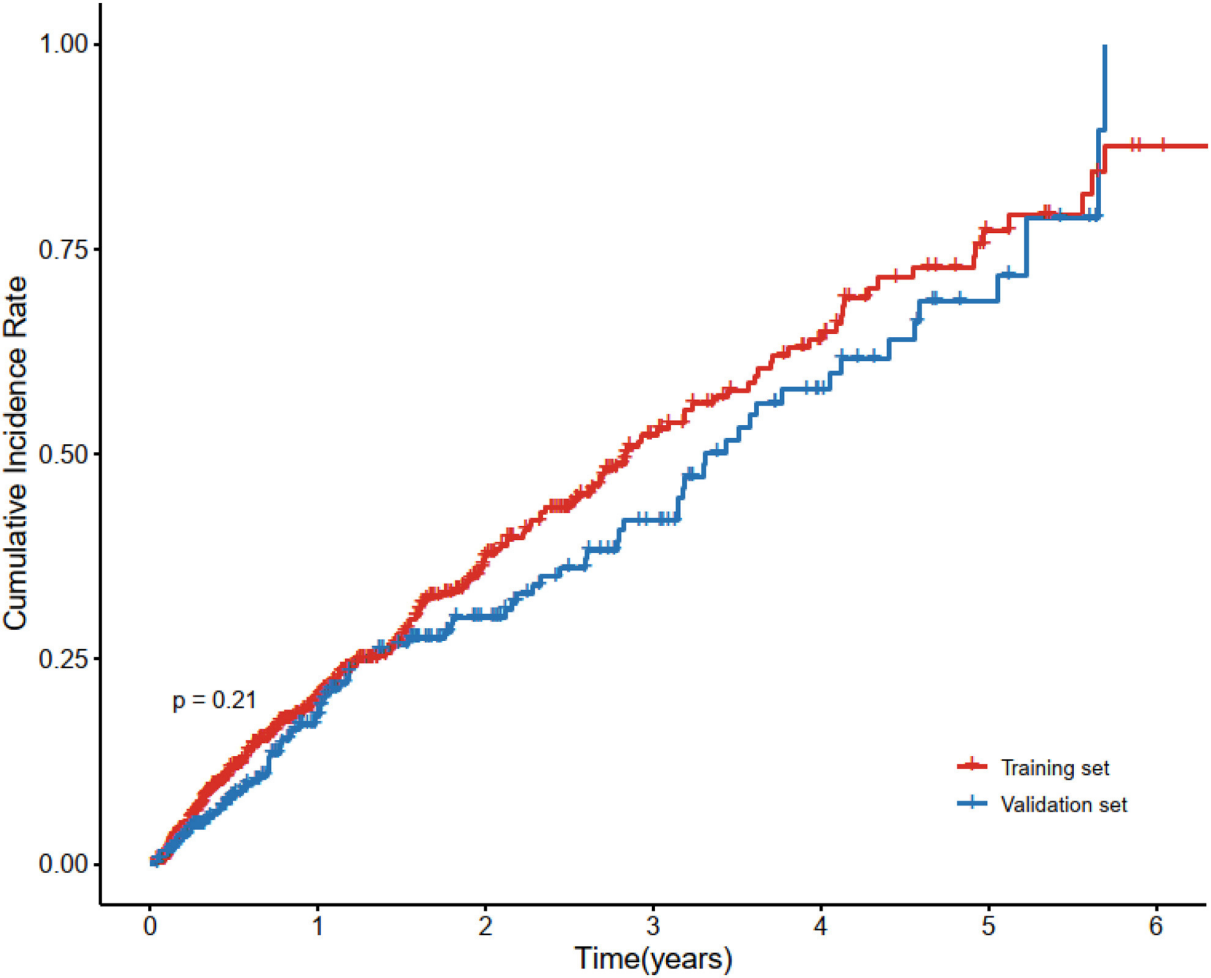
Cumulative incidence curve of cardiovascular disease in the training set and validation set. Cardiovascular disease is the composite of coronary heart disease, cerebrovascular disease, congestive heart failure, and peripheral arterial disease. There was no statistically significant difference between the survival of the two sets using the log-rank test (*p* = 0.21).

A total of 890 patients were included in the final analysis. During a medium follow-up of 10.4 (IQR: 3.8–23.4) months, 284(31.91%) patients required rehospitalization due to subsequent cardiovascular events (128 cases of coronary heart diseases, 98 cerebrovascular diseases, 62 congestive heart failures, and 33 peripheral artery diseases; patients may have had >1 event). The median age for all patients was 52 years (IQR = 43–57), and that was 56 years (IQR = 48–65) for patients who sustained a subsequent CVD. Baseline demographic and clinical characteristics of the included patients, stratified by CVD outcomes are summarized in Supplementary Table 4. Patients were randomly divided into the training set (70%) and the validation set (30%), 606 patients in the training set, and 286 patients in the validation set. There were no statistically significant differences in the variables between the two groups (Table 1). Figure 2 also showed that the cumulative incidence curves of the two sets were no statistically significant difference using the log-rank test (*p* = 0.21).

**Table 1:** Baseline characteristics in the training set and validation set.

	**Total**	**Training set**	**Validation set**	***p*-value**
	**(*n* = 890)**	**(*n* = 623)**	**(*n* = 267)**	
Smoking status				0.474
Never (%)	707 (79.4)	493 (79.1)	214 (80.2)	
Previous (%)	64 (7.2)	42 (6.7)	22 (8.2)	
Current (%)	119 (13.4)	88 (14.1)	31 (11.6)	
Age (years)	52 (45–60)	52 (45–60)	52 (45–60)	0.543
Systolic blood pressure (mmHg)	135 (126–148)	135 (126–148)	135 (125–149)	0.746
Total cholesterol (mmol/L)	4.5 (3.7–5.4)	4.6 (3.7–5.4)	4.5 (3.7–5.3)	0.615
Hemoglobin (g/L)	111 (94–131)	111 (95–130)	111 (92–132)	0.652
high density lipoprotein (mmol/L)	1.1(0.9–1.4)	1.1 (0.8–1.4)	1.1 (0.9–1.4)	0.390
24 h urinary protein (g)	2.7 (0.4–6.3)	2.7 (0.4–6.3)	2.8 (0.4–6.1)	0.296

*Continuous variables are shown as mean (SD) or median (interquartile range) according to the distribution, and categorical variables are shown as frequency (percentage)*.

### Variables Selection

There are 91 baseline clinical variables with at least 70% data completeness as candidate predictors used for LASSO regression. Seven variables, including age, high density lipoprotein (HDL), hemoglobin (Hb), systolic blood pressure (SBP), smoking status, 24 h urinary protein excretion, and total cholesterol (TC), were selected using the lambda with 1 SE of the minimum partial likelihood deviance. These variables are all easily available demographic, clinical characteristics, and laboratory results. Baseline characteristics of the selected variables were presented in Table 2. The results of the univariate analysis and multiple CPH regression analysis showed that each clinical parameter has independent prognostic power (Supplementary Table 5). To explore whether the correlation between variables would have an impact on the model, we applied the correlation-based heat map to calculate the correlation between every two factors. Supplementary Figure 2 revealed that there were few correlations between the chosen variables (all correlation absolute values are <0.5).

**Table 2:** Demographic and clinical characteristics of patients with or without CVD in the dataset.

	**Total**	**CVD**	**NO CVD**	***p*-value**
	**(*n* = 890)**	**(*n* = 284)**	**(*n* = 606)**	
Smoking status				<0.001
Never (%)	707 (79.4)	191 (67.3)	516 (31.5)	
Previous (%)	64 (7.2)	25 (8.8)	39 (4.1)	
Current (%)	119 (13.4)	68 (23.9)	51 (11.2)	
Age (years)	52 (45–60)	56.4 ± 11.7	51 (43–57)	<0.001
Systolic blood pressure (mmHg)	135 (126–148)	142 (131.3–160)	133 (124.8–142)	<0.001
Total cholesterol (mmol/L)	4.5 (3.7–5.4)	4.9 (4.1–5.7)	4.3 (3.6–5.2)	<0.001
Hemoglobin (g/L)	111 (94–131)	107 (91–128)	113 (95.3–133)	0.007
High density lipoprotein (mmol/L)	1.1 (0.9–1.4)	1 (0.8–1.3)	1.1 (0.9–1.4)	0.002
24 h urinary protein (g)	2.7 (0.4–6.3)	3.2 (0.7–7.1)	2.5 (0.4–6)	0.042

*Continuous variables are shown as mean (SD) or median (interquartile range) according to the distribution, and categorical variables are shown as frequency (percentage)*.

### Model Performance

**Figure 3:**
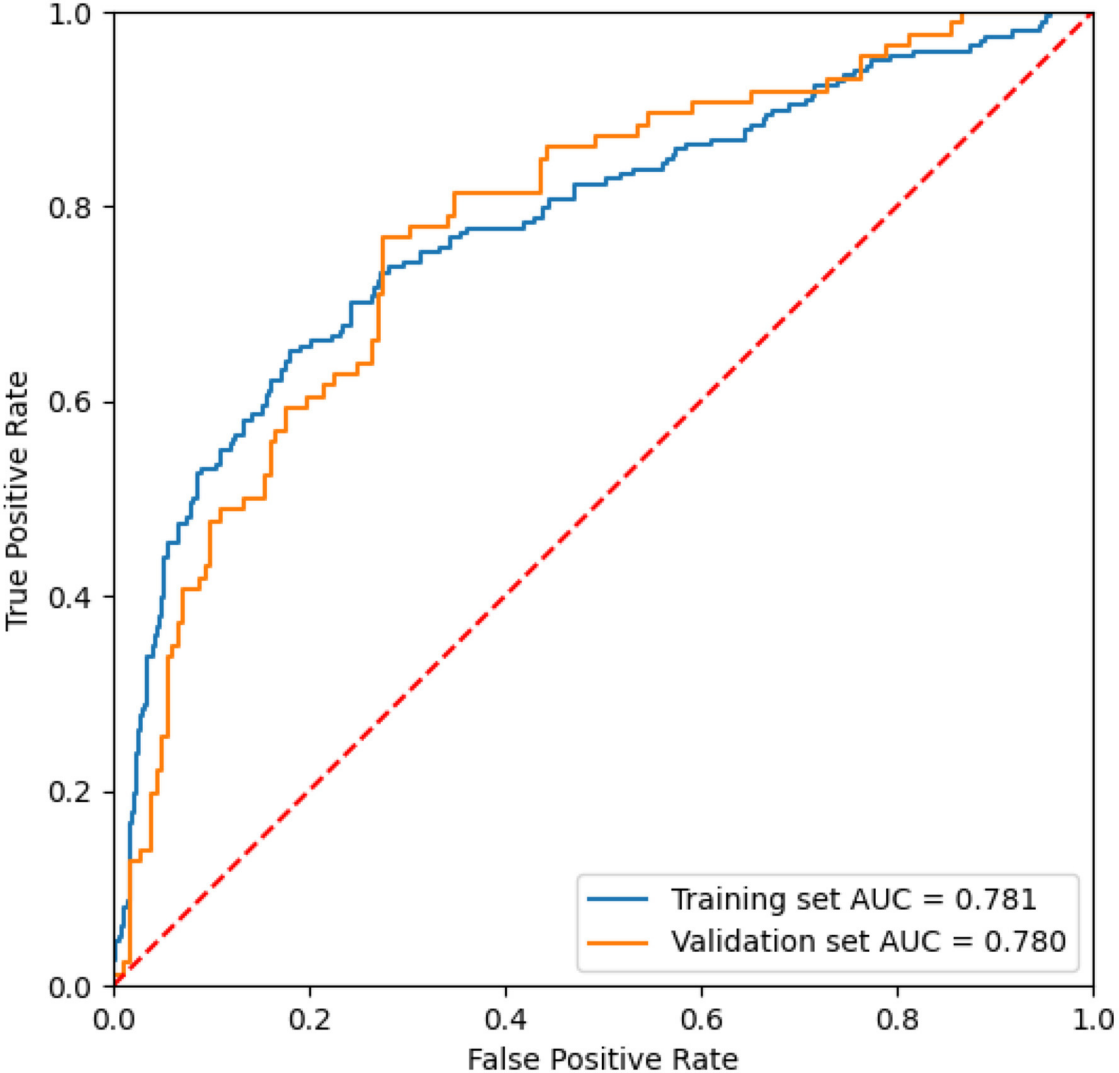
Receiving operating characteristics (ROC) curves for the training set and validation set.

**Figure 4:**
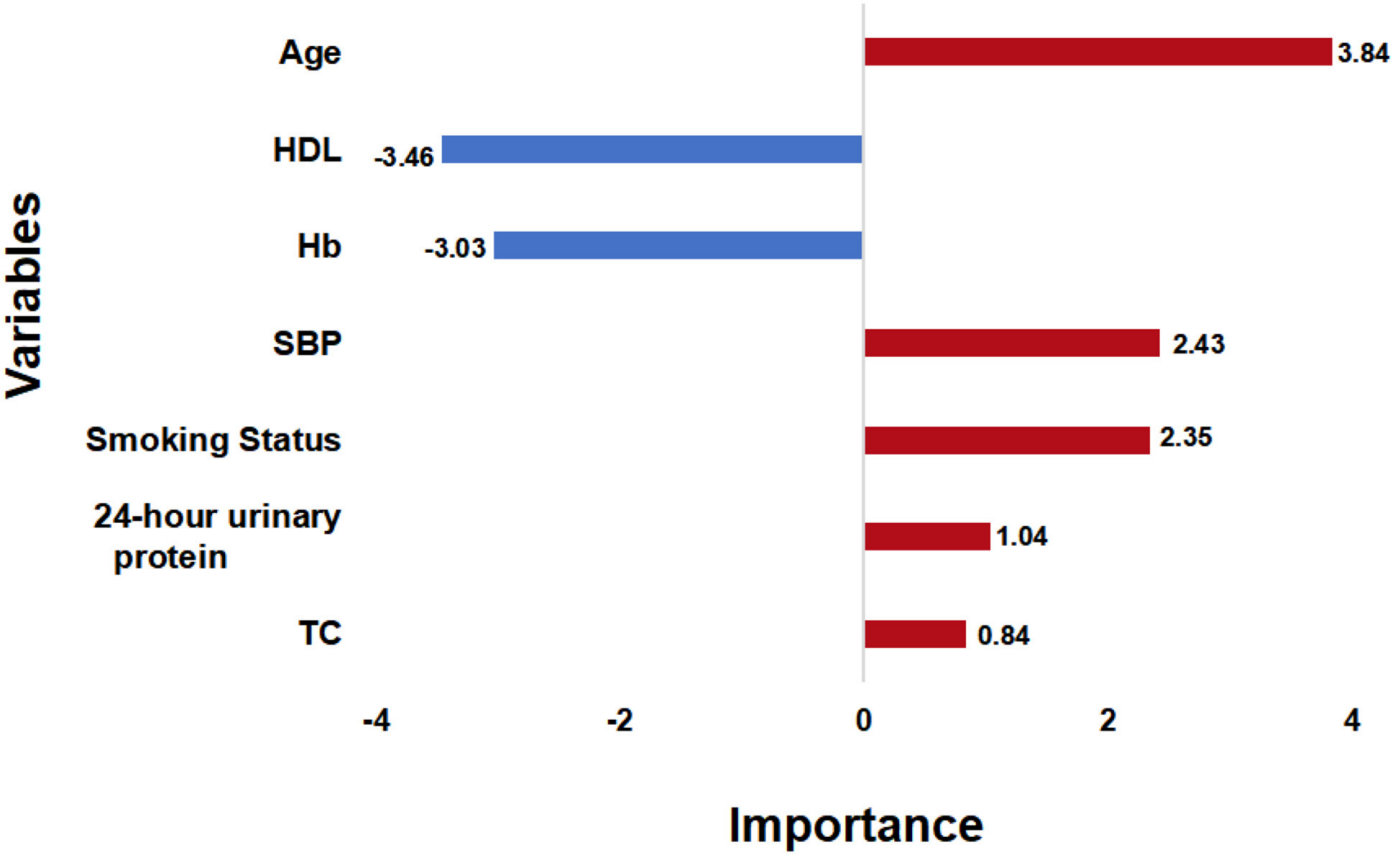
Variable Importance. The importance score of the selected variables is calculated by their weights in the DeepSurv model. SBP, systolic blood pressure; TC, total cholesterol; Hb, hemoglobin; HDL, high density lipoprotein.

After the feature selection process, all seven variables as independent predictors were used for the model development. We use the DeepSurv method to construct survival models to analyze individual CVD outcomes. A three-layer neural network with one input layer, one hidden layer, and one output layer is used to construct the predictive model. We used dropout, batch normalization, and L1 and L2 regularization during training and selected adaptive moment (Adam) as the optimizer and Tanh as the activation function to reduce overfitting. The optimized hyperparameters for DeepSurv are shown in Supplementary Table 6. The 5-fold cross-validation in the training set was performed to prove the robustness of this model, with a mean AUC of 0.779(SD: 0.009), and a C-index of 0.793(SD: 0.012). To evaluate the DeepSurv model discriminative performance, we compared it with the CPH and RSF models using the C-index, AUC, and IBS. Table 3 showed that the DeepSurv model performed best among the three survival models. The C-index of the DeepSurv model in the training and validation sets were 0.796 (95% CI: 0.761–0.831) and 0.796 (95% CI: 0.761–0.831). The AUC were 0.781 (95% CI:0.740–0.822) and 0.780 (95% CI: 0.721–0839) (Figure 3), and the IBS were 0.046 and 0.067 in the training and validation sets respectively. We assess the importance of the variables according to their weight in the DeepSurv model, indicating the univariate contribution to the model. It revealed that older age, lower HDL, lower Hb, higher 24 h urinary protein, smoking, higher SBP, and higher TC were significantly associated with the high risk of CVD (Figure 4). Age, HDL, and Hb were the three main relevant risk factors in the model. Subgroup analysis of four cardiovascular diseases showed that this model performed best in patients with congestive heart failure (Table 4), showed a C-index of 0.874 (95% CI: 0.822–0.826), and the AUC of 0.831 (95%CI: 0.770–0.892). The calibration curves of predicted event probabilities illustrated that this model has higher accuracy in predicting cardiovascular risk at 1 year (Supplementary Figure 3).

**Table 3:** Performance of different models.

	**C-index (95%CI)**		**AUC (95%CI)**		**IBS**	
	**Training set**	**Validation set**	**Training set**	**Validation set**	**Training set**	**Validation set**
DeepSurv	0.796 (0.761–0.831)	0.767 (0.717–0.817)	0.781 (0.740–0.822)	0.780 (0.721–0839)	0.046	0.067
CPH	0.755 (0.719–0.790)	0.745 (0.691–0.800)	0.737 (0.698–0.776)	0.724 (0.659–0.789)	0.177	0.194
RSF	0.721 (0.681–0.761)	0.753 (0.700–0.806)	0.723 (0.680–0.766)	0.765 (0.704–0.826)	0.064	0.148

*Model performance of DeepSurv, RSF, and CPH model in terms of C-index, AUC and IBS. CPH, cox proportional hazards regression; DeepSurv, deep learning-based survival model; RSF, random survival forest model; IBS, integrated brier scores; AUC, the area under the receiver-operator characteristic curve*.

**Table 4:** Subgroup analysis of the performance of different cardiovascular diseases.

**Outcomes**	**C-index (95%CI)**	**AUC (95%CI)**
Coronary heart disease	0.812 (0.768–0.856)	0.771 (0.722–0.820)
Cerebrovascular disease	0.844 (0.802–0.885)	0.806 (0.759–0.853)
Congestive heart failure	0.874 (0.822–0.826)	0.831 (0.770–0.892)
Peripheral artery disease	0.861 (0.789–0.934)	0.726 (0.646–0.806)

*CI, confidence intervals*.

### Risk Stratification

**Figure 5:**
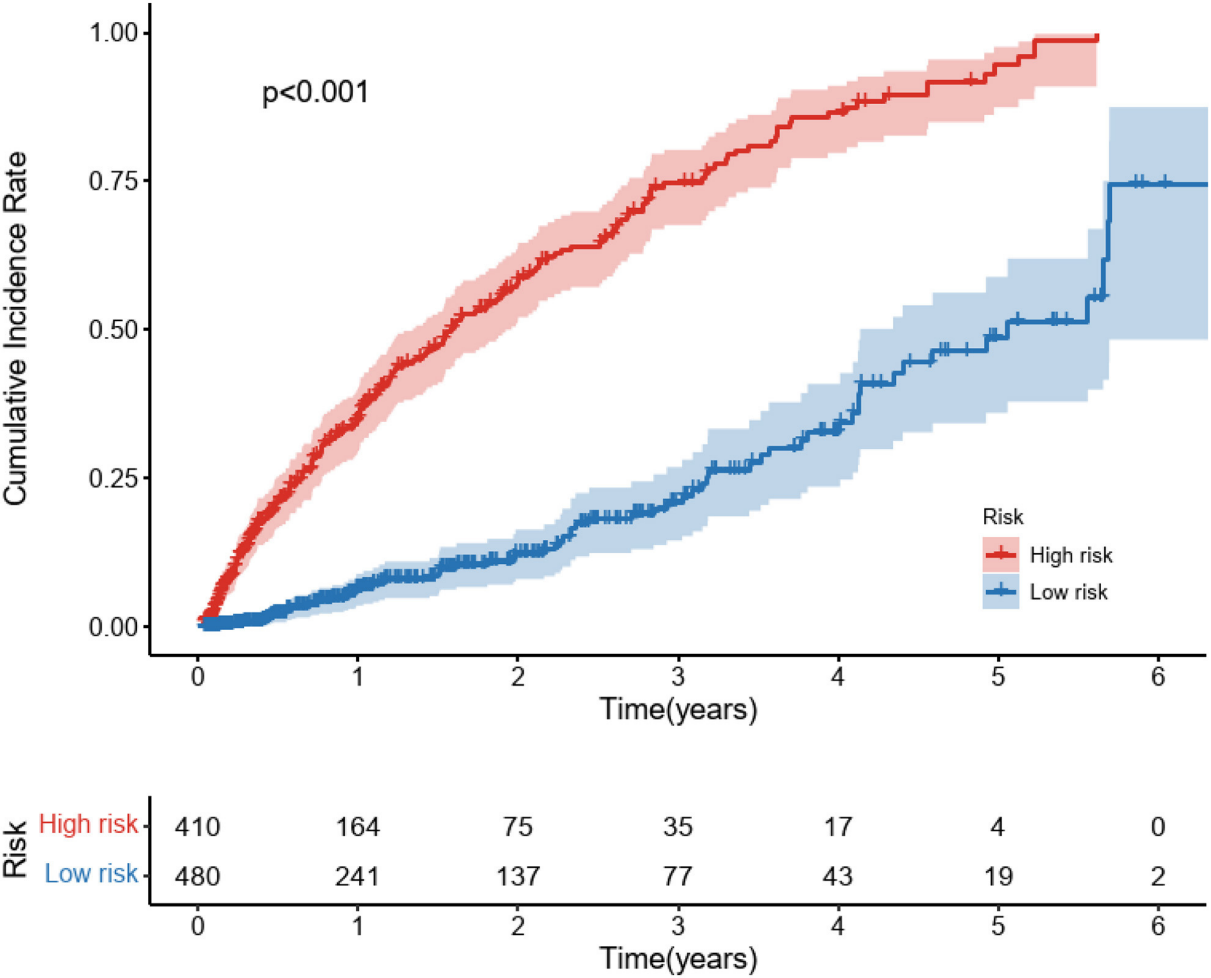
Cumulative Incidence curves for predicted cardiovascular disease among diabetic kidney disease patients in different risk groups. Cardiovascular disease is the composite of coronary heart disease, cerebrovascular disease, congestive heart failure, and peripheral arterial disease. Patients were stratified into a high-risk group and a low-risk group based on the cut-off value of the ROC curve. The *P*-values between the high-risk and low-risk subgroups were calculated by the log-rank test.

Furthermore, after calculating the predicted individual risk score, patients were divided into high-risk and low-risk groups based on the risk cut-off values (the sensitivity of 76.7% and specificity of 72.4%). 410 patients were classified as high risk and 480 patients were classified as low risk group. We plotted the cumulative incidence curves for the two risk subgroups. Figure 5 illustrates that risk stratification based on the DeepSurv model can successfully stratify patients into different risk groups with significant differences (p < 0.01).

### Model Visualization

**Figure 6:**
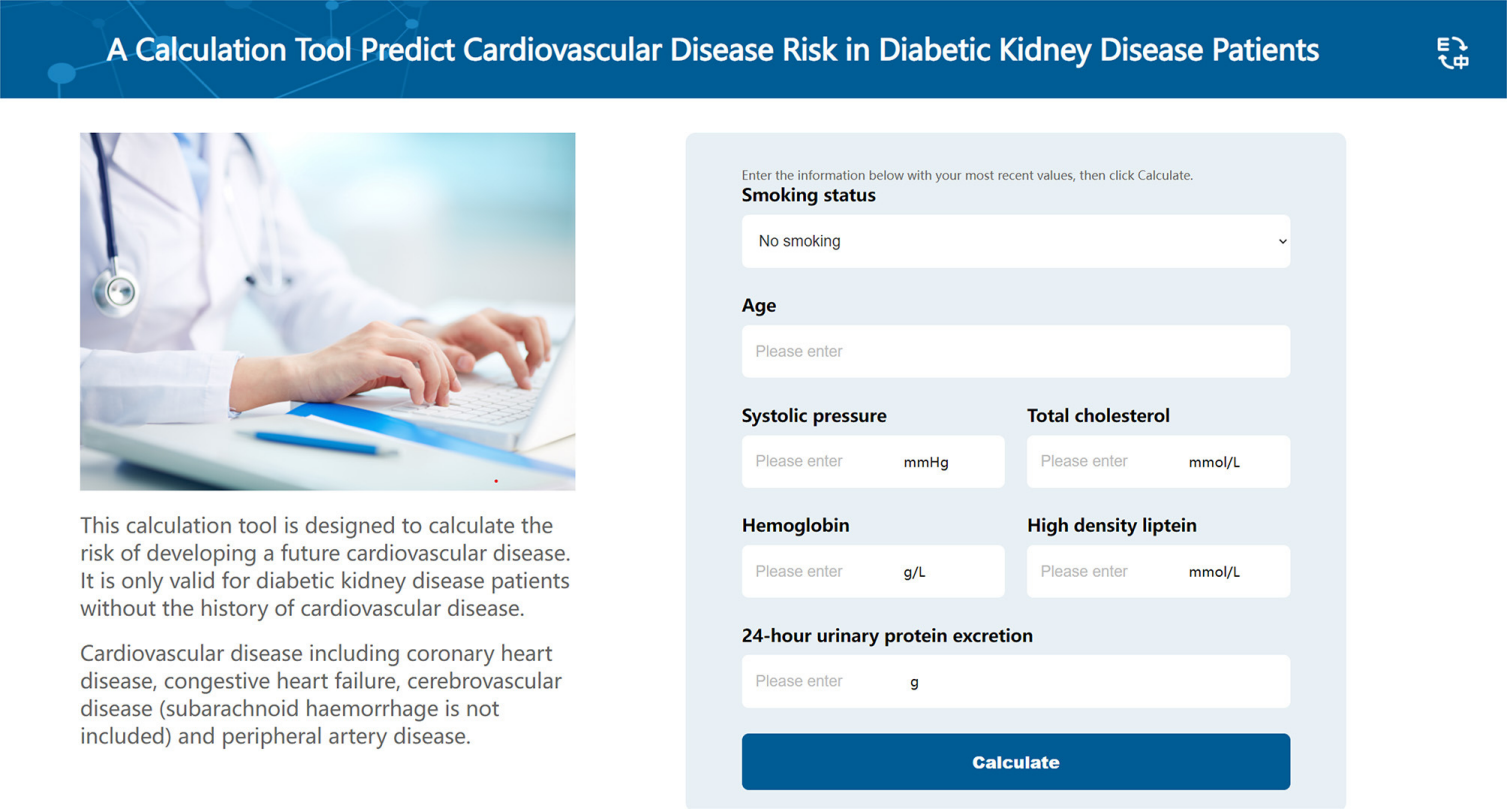
The interface of the online calculation tool. This online calculation tool is used to predict the cardiovascular disease risk among diabetic kidney disease patients.

The best performing model, the DeepSurv model, was used to construct an easy-used online tool to predict CVD risk in DKD patients (http://model.51ehealth.com/). It can calculate the individual CVD risk and monitor the trend of the risk, providing a more intuitive and understandable way to interpret the predictive model. The DeepSurv model was able to plot the predicted Kaplan-Meier survival curves for each patient. Meanwhile, for understanding convenience, we transformed the predicted time-to-event curves output from the model into curves of event incidence rates. DKD patients can input their personal information to get their risk stratification and the incidence of CVD for 1, 3, and 5 year. The interface of this risk calculator is shown in Figure 6. This online tool can also help physicians to choose the appropriate treatments and provide individual recommendations for the individuals to improve outcomes based on the output risk values.

## Discussion

The occurrence and progression of CVD is a crucial factor contributing to poor outcomes in patients with DKD (34). In this study, we developed a deep learning-based predictive model and an online tool to predict the CVD risk in DKD patients. Our model used seven clinical variables, including age, smoking status, SBP, TC, Hb, HDL, and 24 h urinary protein, as independent predictors, and had a promising performance in the validation set. Different from the previous CVD models, our model targets a specific population: DKD patients, which allows it to be applied with greater accuracy. The C-index, ROC curves, and IBS indicated that this deep-learning model had better model discrimination in analyzing patient-individual survival outcomes than the traditional models. Furthermore, the ability to classify patients into different risk groups based on their prognosis may benefit patients by identifying high CVD risk patients and attaching more importance to them. The application of an online tool translates the predictive model into clinical practice, which may be useful for risk calculation and risk monitoring in practical clinical applications. As a result, physicians can determine the most appropriate treatment strategy to implement personalized management based on the results of risk stratification and even improve the CVD outcomes.

This study applied deep learning methods to develop models for prediction and risk stratification of CVD among DKD patients without overfitting been observed, demonstrating that this deep learning-based survival predictive model showed better performance compared to the conventional statistical method. Despite the CPH model being the most widely used approach for survival analysis in analyzing time-to-event survival data (35–37), it has its inherent drawbacks (38). The Cox model assumes the effect of each covariate is proportional and it is unable to properly model non-linearities and interaction effects. Deep-learning methods can learn to solve non-linear and intricate relationships between covariates and individual outcomes efficiently and have advantages in processing large amounts and various types of data (39). But many deep learning methods also have problems with weak interpretation in clinical practice. In this study, DeepSurv methods can combine the deep learning method with the traditional Cox assumption for survival analysis of the non-linear effect using clinical variables to predict the CVD risk of DKD patients. We reveal that this model can significantly improve the prediction performance in terms of the C-index. It not only has good model discrimination but it also can be applied for clinical use for its good interpretability. It can generate predicted Cumulative incidence curves for individuals, thus identifying them into different risk groups. The superior performance of the DeepSurv model demonstrates its ability to handle the complex association of risk factors. In addition, the DeepSurv model has been widely applied to many survival analyses with a favorable prediction value (22, 23, 29). It also can provide a framework on which more datasets can be trained in the future in a broader population.

Numerous risk factors have been proved to be related to the high incidence of CVD in some DKD patients. In this study, we also demonstrated several recognized similar traditional risk predictors, consisting of age, SBP, smoking status, HDL, and TC, in consistent with the previous studies (40). A meta-analysis has shown that these traditional risk factors have been proved in previous classical predictive models based on the general population, including age, blood pressure, smoking status, and cholesterol levels (41). Age is a generally recognized risk factor for CVD (42). During aging, cardiac structural changes and functional dysfunction often occur caused by injury in fundamental cardiomyocytes (43). Hypertension is highly prevalent in chronic kidney disease (CKD) patients, which is widely recognized as a risk factor for the development of CVD (44–47). Reducing blood pressure is an important treatment strategy that not only slows the progression of renal failure but also decreases the risk of cardiovascular disease (48). Smoking is regarded as a crucial and modifiable predictor of the progression of CVD (49). The Study of Heart and Renal Protection (SHARP) found that smoking attributed to the high risk of vascular adverse complications among patients with chronic kidney disease, and may be changed by quitting smoking (50, 51). The potential benefits of cessation are even greater than those of pharmacological treatment for cardiovascular protection (52). There was a strong and inverse correlation between HDL and CVD risk in the DKD population (53). Through reverse cholesterol transport (RCT), HDL can protect against plaque formation and development in the prevention of CVD (54). TC concentration measurement is also proved to be important in the evaluation of CVD risk factors. Besides these traditional risk factors (55), we also found that anemia and high proteinuria play a significant role in the incidence of CVD among DKD patients. Anemia was found significantly associated with the occurrence of CVD events. Anemia can cause changes in ventricular structure (56). Long-term anemia will lead to decreased oxygen capacity and utilization disorders. The compensatory hyperdynamic circulation is needed to maintain normal oxygen supply, resulting in increased cardiac output and left ventricular hypertrophy (57). Anemia often contributes to recurrent and progressive cardiac and renal deterioration, which is also called cardiorenal anemia syndrome (CRAS) (58). Proteinuria, which can reflect kidney lesions, is also a predictive factor of cardiovascular events and mortality (59). A meta-analysis also showed that participants with proteinuria had a higher risk of stroke than non-participants (33). All these included variables were independently correlated with an increased risk of CVD progression. Identifying and increasing awareness of these risk factors for CVD is essential in the early intervention and appropriate treatment of DKD patients. New integrating approaches to prognostic factors could also increase the accuracy of prediction.

There are several limitations to this study. Firstly, this study was a retrospective single-center study. Further prospective research and multicenter datasets are needed to test the generalizability and validity of the model. Secondly, a relatively small number of patients included is also a limitation in our study. Although deep learning methods have advantages in processing data with small sample sizes, Replication in a broader population is needed to confirm the superior predictive potential. Thirdly, our model used only clinical variables. Although the use of these easily accessible variables facilitates the generalization and application of the models, multi-dimensional variables such as medical imaging, omics data, and histopathological information, may also have clinical significance in the occurrence of CVD. Finally, this predictive model is based on the Chinese population, and further validation is needed to verify whether it can be applied to other ethnicities.

In conclusion, we developed and validated a new predictive model with good discrimination to estimate CVD risk among patients with DKD using seven readily available clinical variables. A user-friendly online tool based on this model was developed for clinical implementation and patient surveillance.

## Data Availability Statement

The original contributions presented in the study are included in the article/Supplementary Material, further inquiries can be directed to the corresponding author/s.

## Ethics Statement

The studies involving human participants were reviewed and approved by the Human Scientific Ethics Committee of the First Affiliated Hospital of Zhengzhou University. Written informed consent for participation was not required for this study in accordance with the National Legislation and the Institutional Requirements.

## Author Contributions

JR, JDo, DL, and JDu conceived and designed the study. GL collected the data. JR performed the statistical analysis, explained the results, and wrote the manuscript. JDu discussed the draft manuscript. JDo and ZL revised the manuscript. All the authors approved the submitted version of the manuscript.

## Funding

This study was funded by the Young Scientists Fund of the National Natural Science Foundation of China (Grant No. 82103916), the General Program of the National Natural Science Foundation of China General Project (Grant No. 81970633), and the Major public welfare special projects in Henan Province (Grant No. 201300310600).

## Conflict of Interest

The authors declare that the research was conducted in the absence of any commercial or financial relationships that could be construed as a potential conflict of interest.

## Publisher's Note

All claims expressed in this article are solely those of the authors and do not necessarily represent those of their affiliated organizations, or those of the publisher, the editors and the reviewers. Any product that may be evaluated in this article, or claim that may be made by its manufacturer, is not guaranteed or endorsed by the publisher.
