# Supplementary material for: Prediction and Risk Stratification of Cardiovascular Disease in Diabetic Kidney Disease Patients
Source: Front Cardiovasc Med. 2022 Jun 24;9:923549. doi: 10.3389/fcvm.2022.923549 (PMC9263287; doi:10.3389/fcvm.2022.923549)
Supplement: Supplementary file 1 [file Data_Sheet_1.doc]

**Supplementary Figure 1.** A flowchart for patient selection.


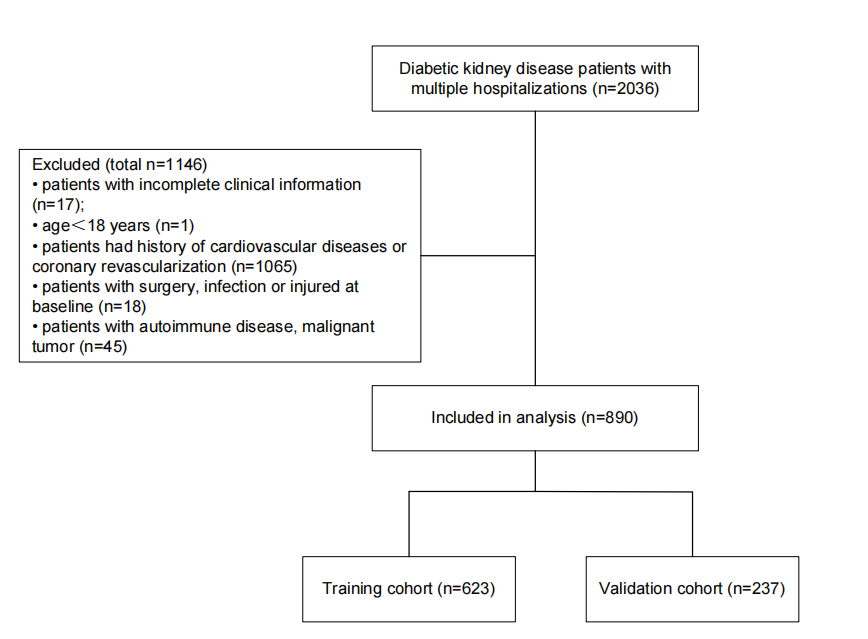


**Supplementary Figure 2.** A Heatmap describing the correlation of the 7 selected variables.


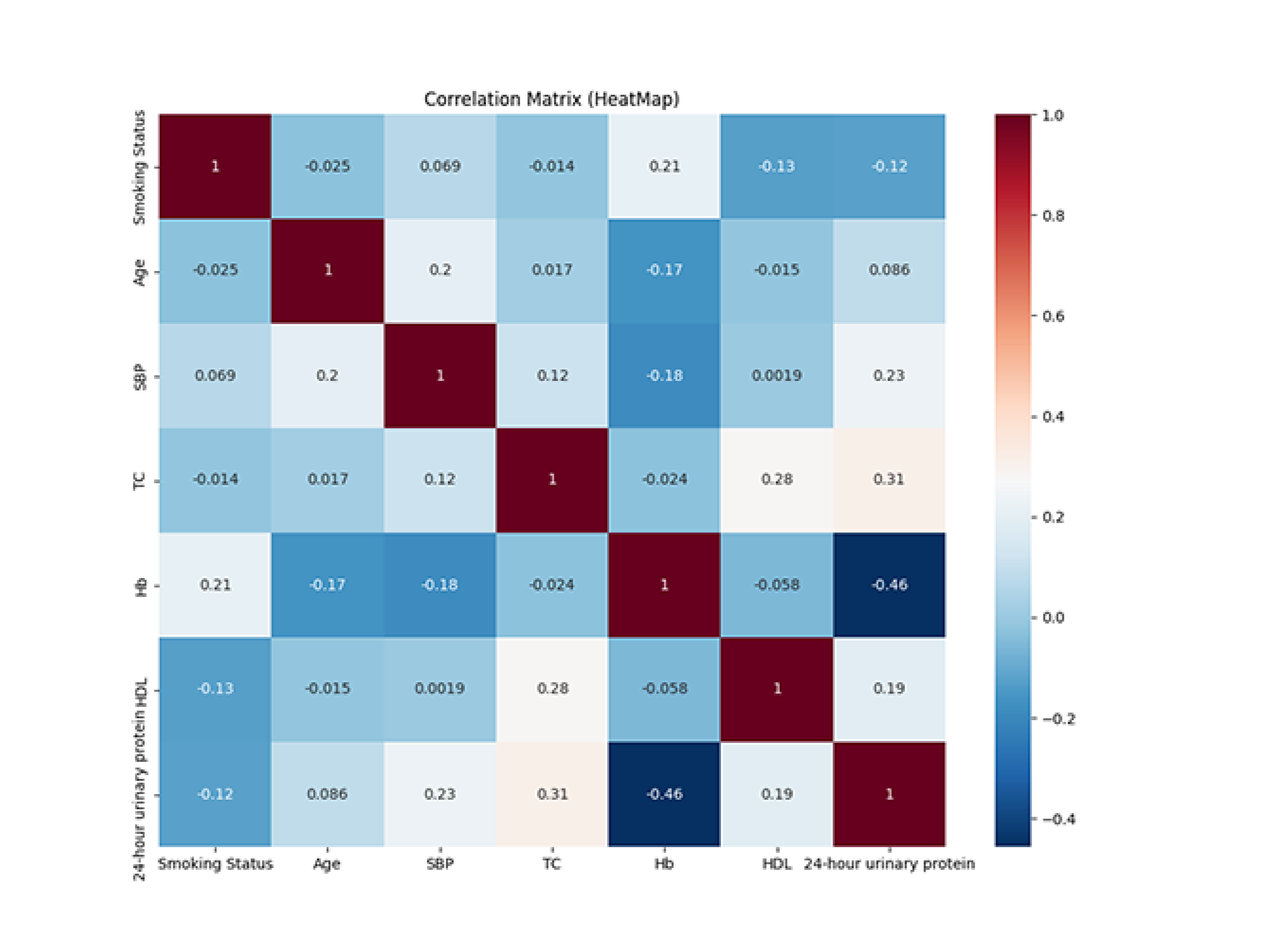


Some negatively related variables are represented in blue, and positively related variables are represented in red. Lager absolute values and darker colors mean higher correlation.

**Supplementary Figure 3:** The Calibration curve for predicting individuals’ risk.


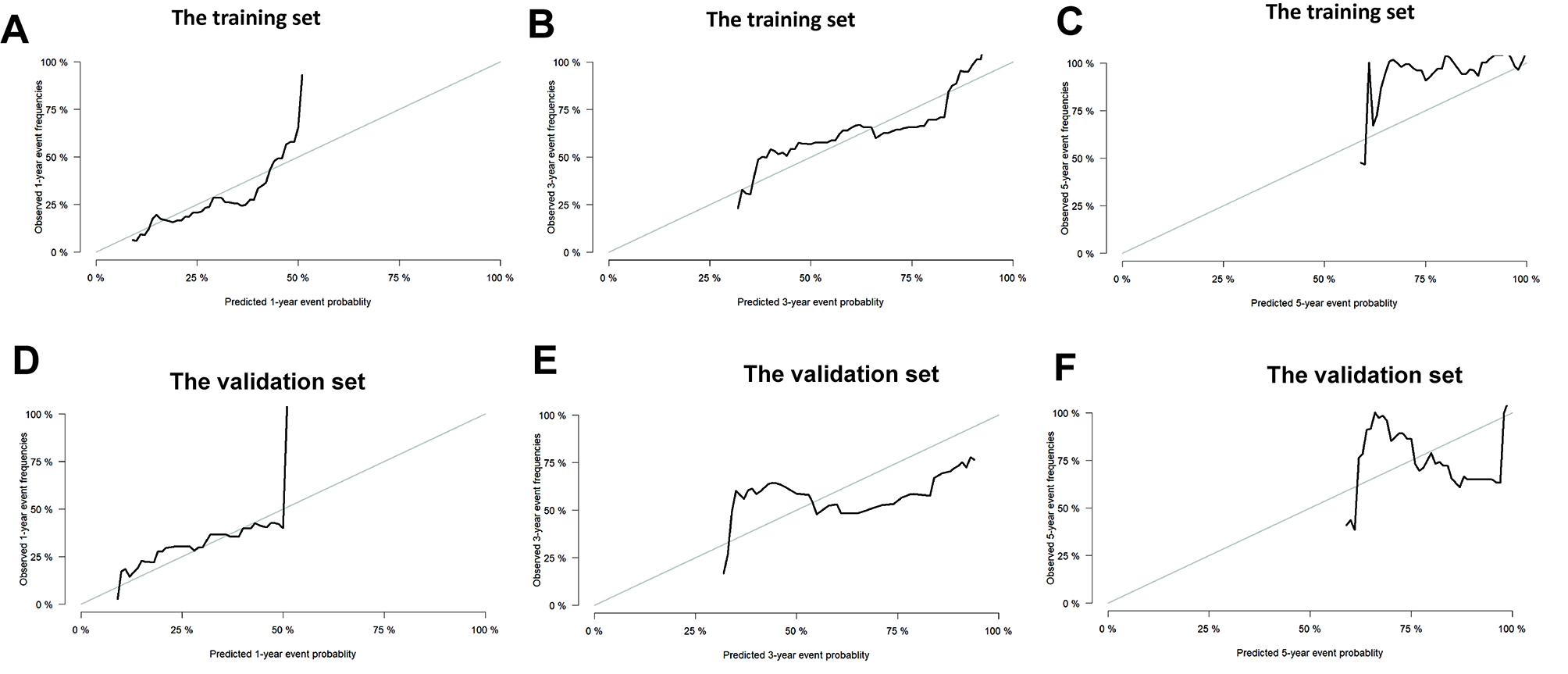


The calibration curve for predicting individuals’ risk. (A) at 1-year risk in the training set; (B) at 3-year risk in the training set; (C) at 5-year risk in the training set; (D) at 1-year risk in the validation set; (E) at 3-year risk in the validation set;(F) at 5-year risk in the validation set;

The X-axis is the predicted event probability and the y-axis is the actual event probability.

**Supplementary Table 1.** Definitions of cardiovascular outcomes.

| Outcomes | ICD-10 code |
| --- | --- |
| Coronary heart disease | I20-I25.9 |
| Cerebrovascular disease | I60-I69， G45, G46 |
| Congestive heart failure | I110, I130, I132, I50, I500, I501, I509 |
| Peripheral artery disease | I70.2-I70.8, I73-I73.9 |

ICD-10: the International Classification of Diseases, Tenth Revision**.**

**Supplementary Table 2: The examples of the list of hyperparameters of DeepSurv.**

| num_rounds | LR | LR-decay | activation | optimizer | L1_reg | L2_reg | dropout | C-index_train | C-index_validation |
| --- | --- | --- | --- | --- | --- | --- | --- | --- | --- |
| 2100 | 0.1 | 1 | Tanh | Adam | 0.000902693 | 0.0002204260 | 0.9 | 0.795890 | 0.766940 |
| 1600 | 0.9 | 0.9999 | ReLU | Adam | 0.000197341 | 0.000819101 | 0.8 | 0.5 | 0.5 |
| 1700 | 0.4 | 1 | ReLU | Adam | 0.000908109 | 0.000820706 | 1 | 0.749601 | 0.643486 |
| 1800 | 0.7 | 1 | Tanh | SGD | 0.000325032 | 0.000155697 | 0.9 | 0.790237 | 0.744683 |
| 1900 | 0.4 | 1 | Tanh | SGD | 0.000605739 | 0.000440276 | 1 | 0.786277 | 0.730586 |
| 2100 | 0.2 | 1 | ReLU | SGD | 0.000360573 | 0.000209182 | 0.9 | 0.789113 | 0.712385 |
| 1600 | 0.1 | 1 | ReLU | SGD | 0.000506347 | 0.000466918 | 1 | 0.792465 | 0.738253 |
| 1800 | 0.6 | 0.9999 | Tanh | Adam | 0.000982089 | 0.000147493 | 1 | 0.820063 | 0.719408 |
| 1800 | 0.2 | 1 | Tanh | Adam | 0.00096163 | 0.00018428 | 0.9 | 0.792194 | 0.750025 |
| 1900 | 0.5 | 0.9999 | ReLU | Adam | 0.000451803 | 0.000839062 | 1 | 0.5 | 0.5 |
| 1900 | 0.4 | 1 | Tanh | SGD | 7.85E-05 | 0.000971874 | 0.9 | 0.784855 | 0.752102 |
| 1700 | 0.9 | 0.9999 | ReLU | SGD | 0.000678583 | 0.000315831 | 0.9 | 0.782472 | 0.718518 |
| 1600 | 0.8 | 0.9999 | ReLU | SGD | 0.000785175 | 0.000919856 | 1 | 0.796914 | 0.710901 |
| 1900 | 0.8 | 0.9999 | ReLU | Adam | 2.26E-05 | 0.000920835 | 0.8 | 0.5 | 0.5 |
| 2000 | 0.9 | 1 | ReLU | SGD | 0.000301891 | 0.000691546 | 0.8 | 0.763835 | 0.719853 |
| 1700 | 0.1 | 1 | ReLU | Adam | 0.00096568 | 0.000490269 | 0.9 | 0.782010 | 0.716045 |
| 1500 | 0.8 | 1 | Tanh | SGD | 0.000576137 | 0.000926279 | 0.9 | 0.787084 | 0.755070 |
| 1800 | 0.9 | 0.9999 | Tanh | SGD | 0.000928534 | 0.000592221 | 0.9 | 0.785181 | 0.741517 |
| 1500 | 0.2 | 0.9999 | ReLU | SGD | 0.000751909 | 0.000610359 | 1 | 0.792755 | 0.721189 |
| 1600 | 0.7 | 0.9999 | Tanh | Adam | 0.000701991 | 0.000796352 | 1 | 0.778830 | 0.738599 |
| 1600 | 0.9 | 0.9999 | ReLU | SGD | 0.000566298 | 8.86E-05 | 1 | 0.5 | 0.5 |
| 1500 | 0.3 | 1 | Tanh | SGD | 0.000552893 | 4.43E-05 | 0.9 | 0.772388 | 0.751410 |
| 1500 | 0.3 | 1 | Tanh | SGD | 0.000566298 | 8.86E-05 | 0.9 | 0.778803 | 0.74834 |
| 1500 | 0.3 | 1 | Tanh | SGD | 0.000608088 | 4.45E-05 | 0.9 | 0.779020 | 0.751410 |
| 1500 | 0.8 | 1 | Tanh | SGD | 0.000277243 | 0.000364109 | 0.9 | 0.787048 | 0.749134 |
| 1500 | 0.3 | 1 | Tanh | SGD | 0.000520537 | 5.78E-07 | 0.9 | 0.778893 | 0.751311 |
| 1500 | 0.3 | 1 | Tanh | SGD | 0.000156349 | 1.97E-05 | 0.9 | 0.779871 | 0.748738 |
| 2000 | 0.3 | 1 | Tanh | SGD | 0.000125524 | 0.000304504 | 0.8 | 0.767441 | 0.739934 |
| 2100 | 0.3 | 1 | Tanh | SGD | 0.000230036 | 2.08E-05 | 0.9 | 0.778784 | 0.746364 |
| 1500 | 1 | 1 | Tanh | Adam | 0.00015245 | 0.000115686 | 0.8 | 0.5 | 0.5 |
| 1500 | 0.6 | 1 | Tanh | SGD | 0.000375714 | 5.51E-06 | 0.9 | 0.778132 | 0.733801 |
| 2100 | 0.7 | 1 | Tanh | SGD | 8.80E-06 | 0.000390192 | 0.9 | 0.785743 | 0.750025 |
| 1500 | 0.3 | 1 | Tanh | SGD | 0.000209202 | 0.000267295 | 0.9 | 0.773801 | 0.755564 |
| 2000 | 0.5 | 1 | Tanh | Adam | 0.000188111 | 0.000230836 | 0.8 | 0.731725 | 0.684587 |
| 1500 | 0.3 | 1 | Tanh | SGD | 8.59E-05 | 0.000284143 | 0.9 | 0.779672 | 0.749629 |
| 1500 | 0.3 | 1 | Tanh | SGD | 0.000250796 | 0.000112942 | 0.9 | 0.779020 | 0.752003 |
| 1700 | 0.1 | 1 | Tanh | SGD | 0.000257312 | 0.000545875 | 0.9 | 0.762983 | 0.738451 |
| 2100 | 1 | 1 | Tanh | Adam | 0.000341809 | 0.000418174 | 0.8 | 0.5 | 0.5 |
| 1800 | 0.4 | 1 | ReLU | Adam | 0.000401538 | 0.000105148 | 0.9 | 0.5 | 0.5 |
| 1500 | 0.6 | 1 | Tanh | SGD | 0.000314283 | 0.00016084 | 0.9 | 0.780669 | 0.736571 |

Abbreviation: LR, Learning rate; LR-decay, Learning rate-decay; ReLU, Rectified Linear Unit; Adam, adaptive moment estimation; SGD, Stochastic Gradient Descent.

**Supplementary Table 3. TRIPOD Checklist for Prediction Model.**

| **Section/Topic** | **Item** | **Checklist Item** | **Page** |
| --- | --- | --- | --- |
| **Title and abstract** | | | |
| Title | 1 | Identify the study as developing and/or validating a multivariable prediction model, the target population, and the outcome to be predicted. | 1 |
| Abstract | 2 | Provide a summary of objectives, study design, setting, participants, sample size, predictors, outcome, statistical analysis, results, and conclusions. | 2 |
| **Introduction** | | | |
| Background and objectives | 3a | Explain the medical context (including whether diagnostic or prognostic) and rationale for developing or validating the multivariable prediction model, including references to existing models. | 3 |
|  | 3b | Specify the objectives, including whether the study describes the development or validation of the model or both. | 3-4 |
| **Methods** | | | |
| Source of data | 4a | Describe the study design or source of data (e.g., randomized trial, cohort, or registry data), separately for the development and validation data sets, if applicable. | 4 |
|  | 4b | Specify the key study dates, including the start of accrual; end of accrual; and, if applicable, end of follow-up. | 4 |
| Participants | 5a | Specify key elements of the study setting (e.g., primary care, secondary care, general population) including number and location of centres. | 4 |
|  | 5b | Describe eligibility criteria for participants. | 4 |
|  | 5c | Give details of treatments received, if relevant. | Not applicable |
| Outcome | 6a | Clearly define the outcome that is predicted by the prediction model, including how and when assessed. | 4, Supp Table 1 |
|  | 6b | Report any actions to the blind assessment of the outcome to be predicted. | Not applicable |
| Predictors | 7a | Clearly define all predictors used in developing or validating the multivariable prediction model, including how and when they were measured. | 4, Table 1, Table 2 |
|  | 7b | Report any actions to the blind assessment of predictors for the outcome and other predictors. | Not applicable |
| Sample size | 8 | Explain how the study size was arrived at. | 4 |
| Missing data | 9 | Describe how missing data were handled (e.g., complete-case analysis, single imputation, multiple imputation) with details of any imputation method. | 4 |
| Statistical analysis methods | 10a | Describe how predictors were handled in the analyses | 4-5 |
|  | 10b | Specify type of model, all model-building procedures (including any predictor selection), and method for internal validation. | 4-5 |
|  | 10d | Specify all measures used to assess model performance and, if relevant, to compare multiple models. | 4-5 |
| Risk groups | 11 | Provide details on how risk groups were created, if done | 5 |
| **Results** | | | |
| Participants | 13a | Describe the flow of participants through the study, including the number of participants with and without the outcome and, if applicable, a summary of the follow-up time. A diagram may be helpful. | Supp Figure 1 |
|  | 13b | Describe the characteristics of the participants (basic demographics, clinical features, available predictors), including the number of participants with missing data for predictors and outcome. | Table 1, Table 2 |
| Model development | 14a | Specify the number of participants and outcome events in each analysis. | Table 1 |
|  | 14b | If done, report the unadjusted association between each candidate predictor and outcome. | Table 2 |
| Model specification | 15a | Present the full prediction model to allow predictions for individuals (i.e., all regression coefficients, and model intercept or baseline survival at a given time point) | 6-7 |
|  | 15b | Explain how to the use the prediction model. | 7-8 |
| Model performance | 16 | Report performance measures (with CIs) for the prediction model. | 6, Table 3 |
| **Discussion** | | | |
| Limitations | 18 | Discuss any limitations of the study (such as nonrepresentative sample, few events per predictor, missing data). | 9-10 |
| Interpretation | 19b | Give an overall interpretation of the results, considering objectives, limitations, and results from similar studies, and other relevant evidence. | 8-9 |
| Implications | 20 | Discuss the potential clinical use of the model and implications for future research | 8-9 |
| **Other information** | | | |
| Supplementary information | 21 | Provide information about the availability of supplementary resources, such as study protocol, Web calculator, and data sets. | 7, Figure 6 |
| Funding | 22 | Give the source of funding and the role of the funders for the present study. | 9 |

**Supplementary Table 4.** All Clinical characteristics of the dataset.

|  | Total (n=890) | CVD (n=289) | NO CVD (n=606) |
| --- | --- | --- | --- |
| Sex |  |  |  |
| Female (%) | 557 (62.6) | 174 (61.3) | 383 (28.7) |
| Male (%) | 333 (37.4) | 110 (38.7) | 223 (18.2) |
| Family history of cardiovascular diseases |  |  |  |
| No (%) | 753 (84.6) | 234 (82.4) | 519 (38.6) |
| Yes (%) | 137 (15.4) | 50 (17.6) | 87 (8.3) |
| Types of diabetes |  |  |  |
| Type 1 diabetes (%) | 58 (6.5) | 12 (4.2) | 46 (2.0) |
| Type 2 diabetes (%) | 832 (93.5) | 272 (95.8) | 560 (44.9) |
| Diabetic retinopathy |  |  |  |
| No (%) | 585 (65.7) | 169 (59.5) | 416 (27.9) |
| Yes (%) | 305 (34.3) | 115 (40.5) | 190 (19) |
| Diabetic peripheral angiopathy |  |  |  |
| No (%) | 750 (84.3) | 227 (79.9) | 523 (37.5) |
| Yes (%) | 140 (15.7) | 57 (20.1) | 83 (9.4) |
| Nation |  |  |  |
| the Han nationality (%) | 885 (99.4) | 282 (99.3) | 603 (46.5) |
| the Hui nationality (%) | 5 (0.6) | 2 (0.7) | 3 (0.3) |
| Atrial fibrillation |  |  |  |
| No (%) | 886 (99.6) | 282 (99.3) | 604 (46.5) |
| Yes (%) | 4 (0.4) | 2 (0.7) | 2 (0.3) |
| Ketone body |  |  |  |
| -(%) | 859 (96.5) | 273 (96.1) | 586 (45) |
| +(%) | 31 (3.5) | 11 (3.9) | 20 (1.8) |
| **Smoking history** |  |  |  |
| Never (%) | 707 (79.4) | 191 (67.3) | 516 (31.5) |
| Previous (%) | 64 (7.2) | 25 (8.8) | 39 (4.1) |
| Current (%) | 119 (13.4) | 68 (23.9) | 51 (11.2) |
| Drinking history |  |  |  |
| Never (%) | 739 (83.0) | 221 (77.8) | 518 (36.5) |
| Previous (%) | 57 (6.4) | 24 (8.5) | 33 (4.0) |
| Current (%) | 94 (10.6) | 39 (13.7) | 55 (6.4) |
| Blood pressure |  |  |  |
| Normotension (%) | 80 (9.0) | 20 (7.0) | 60 (3.3) |
| Prehypertension (%) | 390 (43.8) | 90 (31.7) | 300 (14.9) |
| Hypertension stage 1 (%) | 281 (31.6) | 94 (33.1) | 187 (15.5) |
| Hypertension stage 2 (%) | 107 (12.0) | 62 (21.8) | 45 (10.2) |
| Hypertension stage 3 (%) | 32 (3.6) | 16 (5.6) | 14 (2.6) |
| Chronic kidney disease staging |  |  |  |
| Stage 1 (%) | 273 (30.7) | 67 (23.6) | 206 (11.1) |
| Stage 2 (%) | 126 (14.2) | 36 (12.7) | 90 (5.9) |
| Stage 3 (%) | 149 (16.7) | 48 (16.9) | 101 (7.9) |
| Stage 4 (%) | 130 (14.6) | 54 (19.0) | 76 (8.9) |
| Stage 5 (%) | 212 (23.8) | 79 (27.8) | 133 (13) |
| Urine protein (qualitative) |  |  |  |
| - (%) | 206 (23.1) | 60 (21.1) | 146 (9.9) |
| + (%) | 122 (13.7) | 44 (15.5) | 78 (7.3) |
| 2+ (%) | 177 (19.9) | 54 (19.0) | 123 (8.9) |
| 3+ (%) | 267 (30) | 85 (29.9) | 182 (14.0) |
| 4+ (%) | 118 (13.3) | 41 (14.4) | 77 (6.8) |
| Urine occult blood (qualitative) |  |  |  |
| - (%) | 428 (48.1) | 158 (55.6) | 280 (26.1) |
| + (%) | 230 (25.8) | 72 (25.4) | 158 (11.9) |
| 2+ (%) | 128 (14.4) | 39 (13.7) | 89 (6.4) |
| 3+ (%) | 104 (11.7) | 25 (8.8) | 79 (4.1) |
| Urine glucose (qualitative) |  |  |  |
| - (%) | 415 (46.6) | 143 (50.4) | 272 (23.6) |
| + (%) | 115 (12.9) | 35 (12.3) | 80 (5.8) |
| 2+ (%) | 108 (12.1) | 33 (11.6) | 75 (5.4) |
| 3+ (%) | 211 (23.7) | 59 (20.8) | 152 (9.7) |
| 4+ (%) | 41 (4.6) | 14 (4.9) | 27 (2.3) |
| **Age (years)** | 52 (45-60) | 56.4±11.7 | 51 (43-57) |
| Weight (kg) | 70.5 (61-80) | 71.1±12.9 | 71 (61-80) |
| **Systolic blood pressure** (mmHg) | 135 (126-148) | 142 (131.3-160) | 133 (124.8-142) |
| Diastolic blood pressure (mmHg) | 80 (75.8-89) | 83 (76-90) | 80 (75-89) |
| Total protein (g/L) | 60.7 (52.2-67.2) | 60.8 (52.5-67.8) | 60.7 (52.2-67) |
| **Total cholesterol (mmol/L)** | 4.5 (3.7-5.4) | 4.9 (4.1-5.7) | 4.3 (3.6-5.2) |
| Neutrophil count (10^9/L) | 4.2±1.6 | 4.2±1.6 | 4.2±1.6 |
| Neutrophil percentage (%) | 62.1 (55.8-68.9) | 60.7 (55.1-68.5) | 62.6 (56-69.3) |
| direct bilirubin (μmol/L) | 2.5 (1.7-3.7) | 2.4 (1.7-3.8) | 2.5 (1.8-3.7) |
| Red blood cells count (10^9/L) | 0 (0-0) | 0 (0-0) | 0 (0-0) |
| Red blood cells percentage (%) | 0 (0-0) | 0 (0-0) | 0 (0-0) |
| Plateletocrit (%) | 0.2 (0.1-0.2) | 0.2 (0.1-0.2) | 0.2 (0.1-0.2) |
| platelet count (10^9/L) | 200 (165-251) | 196 (165.3-241.5) | 202 (164.8-258.3) |
| Platelet distribution width (fL) | 16.8 (16.4-17.2) | 16.9 (16.5-17.3) | 16.8 (16.4-17.2) |
| **Hemoglobin (g/L)** | 111(94-131) | 107(91-128) | 113 (95.3-133) |
| Fibrinogen (g/L) | 3.6 (2.9-4.3) | 3.6 (2.9-4.2) | 3.7 (2.9-4.3) |
| Bacteria (/ul) | 1 (0-26.1) | 2 (0-35.1) | 1 (0-21) |
| Hyaline cast count (/μL) | 0 (0-0) | 0 (0-0) | 0 (0-0) |
| Glycosylated hemoglobin (%) | 7.6 (6.5-9.2) | 7.8 (6.6-9.7) | 7.6 (6.4-9) |
| Eosinophil count (10^9/L) | 0.1 (0.1-0.2) | 0.1 (0.1-0.2) | 0.1 (0.1-0.2) |
| Eosinophil percentage (%) | 2.2 (1.2-3.4) | 2.3 (1.3-3.6) | 2.1 (1.2-3.4) |
| Basophil count (10^9/L) | 0.5 (0.4-0.7) | 0.5 (0.4-0.7) | 0.5 (0.4-0.7) |
| Basophil percentage (%) | 0 (0-0.1) | 0 (0-0.1) | 0 (0-0.1) |
| Estimated glomerular filtration rate (mL/min/1.73m^2) | 50.2 (16.4-96.8) | 37.2 (13.1-89.1) | 59.2 (17.7-99.9) |
| Globulin (g/L) | 24.3 (21.3-27.6) | 25.0±5.0 | 24.2 (21.3-27.5) |
| Glucose (blood) (mmol/L) | 7.1 (5.2-10.5) | 7.1 (5.2-10.4) | 7.1 (5.2-10.6) |
| Mean platelet volume (fl) | 8.5 (7.9-9.3) | 8.5 (7.8-9.3) | 8.5 (7.9-9.4) |
| Mean corpuscular hemoglobin concentration (g/L) | 331 (325-337) | 330(325-336.9) | 331 (325-337) |
| Mean Corpuscular Hemoglobin (pg) | 29.8 (28.7-30.7) | 29.6 (28.8-30.6) | 29.9 (28.7-30.8) |
| Mean corpuscular volume (fl) | 89.7 (86.9-92.8) | 89.8±4.9 | 89.8 (86.9-92.8) |
| Prothrombin time activity (%) | 120 (106.9-135) | 120 (106-136) | 120 (107-133) |
| Thrombin time (s) | 15.4 (14.4-16.4) | 15.4 (14.3-16.6) | 15.4 (14.5-16.3) |
| prothrombin time (s) | 9.8(9.3-10.4) | 9.8(9.2-10.3) | 9.8(9.3-10.5) |
| Acidity | 6(6-6) | 6(6-6) | 6(6-6) |
| Uric acid (μmol/L) | 322(256-385) | 323.6±98.7 | 321(257-385) |
| Urea (mmol/L) | 8.1 (5.6-13.6) | 8.2 (6-13.2) | 8.1(5.4-13.8) |
| Sodium (mmol/L) | 142 (139-144) | 142 (139.9-144) | 142(139-144) |
| Magnesium (mmol/L) | 1 (0.9-1) | 0.9 (0.9-1) | 1 (0.9-1.1) |
| Chloride (mmol/L) | 103.7 (101-106) | 103.9 (100.9-106.4) | 103.3 (101-106) |
| Squamous cell (/μL) | 0.7 (0-3) | 0.7 (0-2) | 1 (0-3) |
| Inorganic phosphate (mmol/L) | 1.3 (1.1-1.5) | 1. 3 (1.1-1.5) | 1.3 (1.1-1.5) |
| Lymphocyte count (10^9/L) | 1.7 (1.2-2.1) | 1.7 (1.3-2.1) | 1.7 (1.2-2.1) |
| Lymphocyte percentage (%) | 26.7±9.4 | 27.1±9.6 | 26.5±9.3 |
| Yeast (/μL) | 0 (0-0) | 0 (0-0) | 0 (0-0) |
| Alkaline phosphatase (U/L) | 72 (58-91) | 73 (61-93) | 71 (58-89) |
| Indirect bilirubin (μmol/L) | 2.8 (1.7-4.4) | 2.8 (1.6-4.7) | 2.7 (1.7-4.4) |
| Kalium (mmol/L) | 4.4 (4-4.8) | 4.4±0.6 | 4.4 (4.1-4.8) |
| Creatinine (μmol/L) | 118 (73-295.5) | 123 (73.3-282.3) | 114 (72.8-299.3) |
| Activated partial thromboplastin time (s) | 31.6 (29.2-34.3) | 32 (29.3-34.9) | 31.5 (29.2-34) |
| Hematokrit (L/L) | 0.3 (0.3-0.4) | 0.3 (0.3-0.4) | 0.3 (0.3-0.4) |
| Red blood cell distribution width (%) | 13.6(13-14.6) | 13.6(13-14.6) | 13.6 (13-14.6) |
| Red blood cell (stool) (/HP) | 0 (0-0) | 0 (0-0) | 0 (0-0) |
| Red blood cell (blood) (10^12/L) | 3.8 (3.2-4.4) | 3.7 (3.2-4.4) | 3.8 (3.2-4.5) |
| Red blood cell (urine) (/μL) | 3 (0-9.1) | 3 (0-9.2) | 3 (0-9.1) |
| International normalized ratio | 0.9 (0.8-0.9) | 0.9 (0.8-0.9) | 0.9 (0.8-0.9) |
| Aspartate aminotransferase (U/L) | 16.8 (13-22) | 16 (13-21) | 17 (13-23) |
| Alanine transaminase (U/L) | 15 (10.8-23) | 14 (10-22) | 15 (11-24) |
| Glutamyl transpeptidase (U/L) | 21 (14-33.3) | 21 (14-36.2) | 21 (14-32) |
| **high density lipoprotein (mmol/L)** | 1.1 (0.9-1.4) | 1 (0.8-1.3) | 1.1 (0.9-1.4) |
| Triglyceride (mmol/L) | 1.7 (1.1-2.5) | 1.7 (1.2-2.4) | 1.6 (1.1-2.5) |
| Calcium (mmol/L) | 2.2 (2.1-2.3) | 2.2 (2.1-2.3) | 2.2 (2.1-2.3) |
| Nonsquamous cell (/μL) | 0 (0-0) | 0 (0-0) | 0 (0-0) |
| Carbon dioxide combining power (mmol/L) | 23.8±3.8 | 23.8±3.8 | 23.8±3.7 |
| low density lipoprotein (mmol/L) | 2.8 (2.1-3.6) | 3 (2.2-3.7) | 2.8 (2.1-3.6) |
| Total bilirubin (μmol/L) | 5.3 (3.6-8.1) | 5.5 (3.4-8.5) | 5.1 (3.7-8) |
| Monocytes count (10^9/L) | 0.5 (0.4-0.6) | 0.5 (0.4-0.6) | 0.5 (0.4-0.6) |
| Monocytes percentage (%) | 7.3 (6.1-8.9) | 7.5 (6.2-9.1) | 7.3 (6.1-8.8) |
| Pathological (/μL) | 0 (0-0) | 0 (0-0) | 0 (0-0) |
| Specific gravity | 1 (1-1) | 1 (1-1) | 1 (1-1) |
| White blood cell count (stool) (/HP) | 0 (0-0) | 0 (0-0) | 0 (0-0) |
| White blood cell count (blood) (10^9/L) | 6.5 (5.3-7.7) | 6.3 (5.2-7.5) | 6.5 (5.4-7.9) |
| White blood cell count (urine) (/μL) | 4 (1-11.2) | 3 (0.7-9) | 4 (1-12) |
| Albumin (g/L) | 36.3 (29.6-41.7) | 35.7±8.3 | 36.2 (29.6-41.9) |
| D-dimer (mg/L) | 0.2 (0.1-0.4) | 0.2 (0.1-0.4) | 0.2 (0.1-0.4) |
| **24h urinary protein (g)** | 2.7 (0.4-6.3) | 3.2 (0.7-7.1) | 2.5 (0.4-6) |
| 24h urine volume (L) | 2 (1.5-2.5) | 2 (1.5-2.5) | 1.9 (1.5-2.5) |

Variables are expressed as mean ± standard deviation, median (±IQR), or frequency (%). (highlighted lines are variables selected by our model). Abbreviation: /HP high power objective.

**Supplementary Table 5.** Univariate and Multivariate analysis.

|  | Univariate | | Multivariate | |
| --- | --- | --- | --- | --- |
|  | HR (95% CI) | P-value | HR (95% CI) | P-value |
| Smoking status | 1.19 (1.08-1.31) | <0.001 | 1.29 (1.17-1.43) | <0.001 |
| Age | 1.66 (1.47-1.87) | <0.001 | 1.56 (1.37-1.77) | <0.001 |
| Systolic blood pressure | 1.47 (1.32-1.64) | <0.001 | 1.19 (1.05-1.34) | 0.006 |
| Total cholesterol | 1.34 (1.21-1.49) | <0.001 | 1.25 (1.12-1.41) | <0.001 |
| Hemoglobin | 0.67 (0.60-0.75) | <0.001 | 0.65 (0.57-0.75) | <0.001 |
| high density lipoprotein | 0.80(0.70-0.92) | 0.002 | 0.73 (0.63-0.85) | <0.001 |
| 24-hour urinary protein | 1.39 (1.25-1.53) | <0.001 | 1.15 (1.01-1.31) | 0.037 |

Abbreviation: HR, hazard ratio; CI, confidence interval.

**Supplementary Table 6. Hyperparameters are used for the DeepSurv model.**

| Bayesian Optimization | LR | LR-decay | optimizer | Activation | L1-reg | L2-reg | dropout |
| --- | --- | --- | --- | --- | --- | --- | --- |
| Hyperparameters | 0.1 | 1 | Adam | Tanh | 9.03e-4 | 2.20e-4 | 0.9 |

Abbreviation: LR, Learning rate; LR-decay, Learning rate-decay; Adam, adaptive moment estimation.
